# Identification of gene mutations associated with type 1 diabetes by next-generation sequencing in affected Palestinian families

**DOI:** 10.3389/fgene.2023.1292073

**Published:** 2024-01-11

**Authors:** Abrar Bawatneh, Alaa Darwish, Hasan Eideh, Hisham M. Darwish

**Affiliations:** ^1^ Molecular Genetics and Genetics Toxicology Program, Faculty of Graduate Studies, Arab American University, Jenin, Palestine; ^2^ Faculty of Health Professions, AlQuds University, Jerusalem, Palestine; ^3^ Layan Medical Center, Ramallah, Palestine; ^4^ Faculty of Allied Medical Sciences, Arab American University, Jenin, Palestine

**Keywords:** type diabetes genetics, gene function, molecular genetic, WES, in born errors of metabolism

## Abstract

**Introduction:** Diabetes Mellitus is a group of metabolic disorders characterized by hyperglycemia secondary to insulin resistance or deficiency. It is considered a major health problem worldwide. T1DM is a result of a combination of genetics, epigenetics, and environmental factors. Several genes have been associated with T1DM, including *HLA*, *INS*, *CTLA4*, and *PTPN22*. However, none of these findings have been based on linkage analysis because it is rare to find families with several diabetic individuals. Two Palestinian families with several afflicted members with variations in the mode of inheritance were identified and selected for this study. This study aimed to identify the putative causative gene(s) responsible for T1DM development in these families to improve our understanding of the molecular genetics of the disease.

**Methods:** One afflicted member from each family was selected for Whole-Exome Sequencing. Data were mapped to the reference of the human genome, and the resulting VCF file data were filtered. The variants with the highest phenotype correlation score were checked by Sanger sequencing for all family members. The confirmed variants were analyzed *in silico* by bioinformatics tools.

**Results:** In one family, the *IGF1R* p.V579F variant, which follows autosomal dominant inheritance, was confirmed and segregated in the family. In another family, the *NEUROD1* p.P197H variant, which follows autosomal recessive inheritance, was positively confirmed and segregated.

**Conclusion:** *IGF1R* p.V579F and *NEUROD1* p.P197H variants were associated with T1DM development in the two inflicted families. Further analysis and functional assays will be performed, including the generation of mutant model cell systems, to unravel their specific molecular mechanism in the disease development.

## 1 Introduction

Diabetes mellitus is a group of metabolic disorders characterized by the presence of hyperglycemia secondary to insulin resistance or deficiency, and it is associated with abnormalities in lipid and protein metabolism and electrolyte disturbances (Kharroubi and Darwish, 2015). Diabetes is considered a great health problem worldwide, with about 425 million adults having been recorded as affected in 2017 (Tremblay and Hamet, 2019). Diabetes mellitus can be classified into type 1 diabetes (T1D), which is characterized by insulin deficiency, type 2 diabetes (T2D), which is characterized by resistance to the action of insulin, gestational diabetes, which develops in women during pregnancy, and Maturity-Onset Diabetes of the Young (MODY), which is characterized by autosomal dominant inheritance and non-dependence on insulin (Anik et al., 2015). The annual health report in Palestine in 2018 reported diabetes health complications as the fifth-ranked cause of death (about 7.5% of all deaths). In 2018, the incidence rate of diabetes mellitus in the West Bank region was reported as 210.7 cases per 100,000 people. There were 2,420 cases among males and 3,135 among females. The mortality rate was 20.4 per 100,000 people (Health annual Palestine report, 2018).

T1D is an autoimmune disease in which insulin is deficient due to the destruction of pancreatic beta cells. There are two forms of T1DM: type 1A, which results from cell-mediated attack and is characterized by the presence of autoantibodies to beta cells, and type 1B, which results from unknown causes and includes unmeasurable autoantibodies or monogenic diabetes (Nyaga et al., 2018; Giwa et al., 2020). Patients are diagnosed in the first few months after birth based on the absence of insulin, detection of C-peptide, and presence of antibodies in the serum. These patients will be treated for life without interruption by insulin and are monitored for several metabolic indicators. T1D is the most common form in children and white persons, accounting for 80% of childhood diabetes in the United States (Redondo et al., 2017). The risk of the disease increases in relatives and siblings and exceeds 70% in identical twins (Bradfield et al., 2011). This clearly implies that T1D is associated with genetic factors. Overall, T1D represents a multifactorial disease that develops from the interplay of genetic, epigenetic, and environmental factors, and it can be affected by age, ethnicity, race, geography, and socioeconomic status (Tremblay and Hamet, 2019).

Genetics are responsible for 80% of hereditary T1D; the HLA region on chromosome 6p21 accounts for 40%–50% of familial T1D. Variation in this region is correlated with the role of autoimmunity in T1D development and can increase its risk or be protective. HLA DR and DQ (part of MCH class II) form alpha-beta heterodimer cell surface antigens and have the strongest association with T1D. The Type 1 Diabetes Genetics Consortium showed that DRB1*0301-DQA1*0501-DQB1*0201, DRB1*0405-DQA1*0301-DQB1*0302, and DRB1*0401-DQA1*0301-DQB*0302 are the most susceptible haplotypes. Whereas, DRB1*1501-DQA1*0102-DQB1*0602, DRB1*1401-DQA1*0101-DQB1*0503, and DRB1*0701-DQA1*0201-DQB1*0303 are the most protective haplotypes (Erlich et al., 2013).

The insulin gene region on chromosome 11 (locus 11p15) constituted the next strongest genetic factor in T1D, and the third associated locus was the cytotoxic T lymphocyte-associated protein 4 (*CTLA4*) gene (Cronin et al., 2017). The protein tyrosine phosphatase non-receptor type 22 (*PTPN22*) gene was reported to be the fourth identified locus in 2004 (Bottini et al., 2004). Later, the interleukin 2 receptor alpha (*IL2RA*) gene (Vella et al., 2005) and the interferon-induced helicase C domain 1 (*IFIH1*) gene on chromosome 2q24.3 (Smyth et al., 2006) were reported as the fifth and sixth associated loci, respectively. In a Japanese study, the *FOX3P* gene, which plays a role in the regulation of T-cell differentiation, was also found to be associated with T1D (Bassuny et al., 2003). Genome-wide association studies were later started to search for further loci associated with T1D (Bakay et al., 2019). They confirmed previous loci and showed evidence of new loci on chromosomes 16p13, 12q13, 12q24, and 18p11. The 16p13 region contains the *KIAA0350* gene, which lies within a 233 kb LD block and is suspected to produce a sugar-binding C-type lectin (Hakonarson et al., 2007; Todd et al., 2007; Concannon et al., 2008). Regions like 12q13 and 12q24 contain many genes in which some of these loci play a role in immune signaling and insulin production, and they are evidenced to be associated with T1D genes such as *ERBB3*, *CYP27B1*, and *SH2B3* (Cooper et al., 2012; Groop and Pociot, 2014). The 18p11 region contains the *PTPN2* gene, which has a function in the immune system and cytokine-induced apoptosis (Groop and Pociot, 2014; Pociot, 2017). Other groups conducted their investigation on 2,496 families inflicted with T1D genotyped with a panel of 6,090 SNPs and reported additional T1D-dependent loci on 21q22.3 in the *UBASH3A* locus (Concannon et al., 2008).

Arab countries have one of the highest incidence and prevalence rates of T1D, which varies from low prevalence (2.54/100,000) in Oman to significantly higher values in Saudi Arabia (29/100,000) (Zayed, 2016). The DRB1*030101-DQB1*0201 haplotypes increased the risk of T1D in Tunisia, Lebanon, and Bahrain, whereas the DRB1*040101-DQB1*0302 haplotype was highly associated with Tunisia and Bahrain but is protective among the Lebanese population (Al-Jenaidi et al., 2005; Stayoussef et al., 2009). The most common HLA haplotypes in Egypt were DRB1*0301-DRB3*0201- DQA1*0501-DQB1*0201 and DRB1*04:02-DQA1*03- DQB1*03:02, while the protective one was DRB1*04:03-DQA1*03DQB1*03:02 (El-Amir et al., 2015). Studies from Morocco showed that Moroccans share some susceptible and protective haplotypes with other populations, such as those from Tunisia, Algeria, Spain, and France, including DRB1*08-DQA1*0401-DQB1*0402 and DRB1*07-DQA1*0201-DQB1*020 haplotypes, respectively (Nadia et al., 2012). Concerning non-HLA genes, several studies reported T1D in Arabs was associated with *PTPN22, CTLA4, IL15, ZAP70, CD3z, CD28, TCRβ*, and *BANK1* (Zayed, 2016). However, no previous studies on the molecular genetics of T1D in Palestine have been reported. We have identified two Palestinian families with several affected members with clear variations in the inheritance pattern of the disease. We hypothesized the presence of putative gene mutations that have a direct role in developing T1DM in these families. Therefore, the objective of this study was to investigate the molecular genetics of T1D development in these families using WES technology. Such genes will provide valuable information to increase our understanding of the molecular genetics basis of Type 1 diabetes and provide tools for early diagnosis and clinical intervention to improve the survival and life quality of these patients before severe complications develop in these individuals.

## 2 Materials and methods

### 2.1 Characteristics of study subjects

**FIGURE 1:**
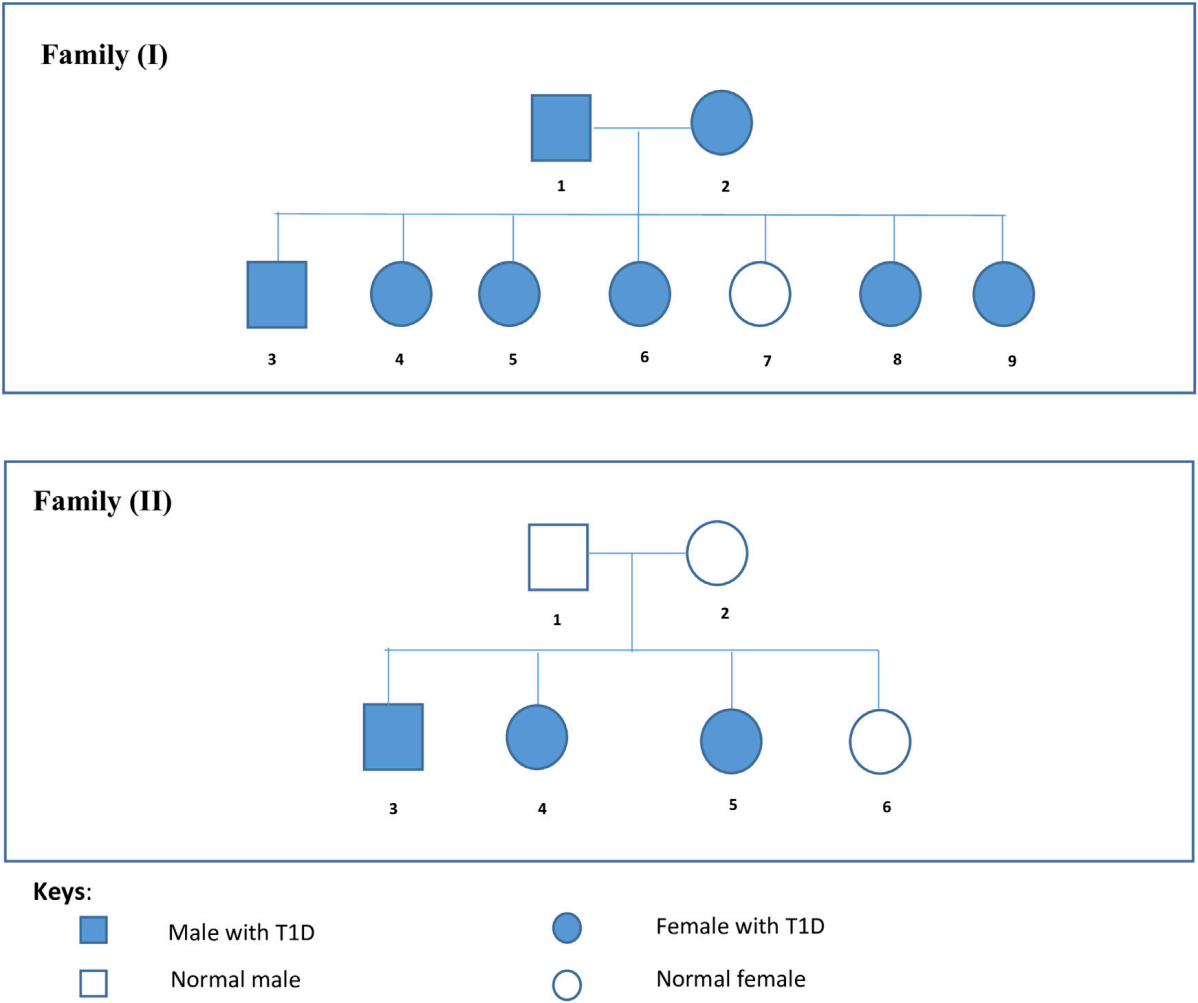
Families’ Pedigrees. Family I is composed of diabetic parents, six diabetic offspring, and one non-diabetic daughter. Family II is composed of non diabetic parents, three diabetic offspring, and a non-diabetic daughter.

Two Palestinian families inflicted with Type 1 diabetes mellitus were included in this study and referred to us by a specialized local Medical Center (Layan Center) in Ramallah, Palestine. They were confirmed to have T1DM based on specific parameters including high serum glucose (above 350 g/dL), high levels of HbA1C (>7%), Low levels of C peptide, the presence of autoantibodies, and insulin dependence. Family I was composed of diabetic parents, six diabetic offspring, and one non-diabetic daughter, indicating the presence of a potentially dominant gene factor. Family II was composed of non-diabetic parents, three diabetic offspring, and a non-diabetic daughter, indicating the presence of a potentially recessive gene mutation. Figure 1 shows the pedigree of both families. Written informed consent was obtained from all participants.

### 2.2 DNA purification

Genomic DNA was extracted from peripheral blood samples according to a commercially available kit (Master PureTM Genomic DNA Purification Kit, Epicenter Technology Co. Cat.No.MG71100) (Mcd, 2012). Extracted DNA was checked for purity, concentration size, and integrity using Nanodrop spectrophotometer and Agarose Gel Electrophoresis and stored at −30°C.

### 2.3 Library preparation and exome sequencing

Samples were subjected to whole-exome sequencing using Illumina Nextseq550 according to the manufacturer’s instructions (Illumina, 2021).

### 2.4 NGS data analysis

Fastq paired-end reads were mapped to the reference human genome version GRCh38 using the Burrows-Wheeler Alignment Tool with the BWA-MEM algorithm. The mapped reads in the bam file were filtered for the following two criteria; first, we retained only paired reads for which both the forward and the reverse read have been mapped to the reference successfully using Samtools. Second, PCR duplicates were removed using the RmDup tool. Filtered mapped reads were then used to call the variants using the FreeBayes variant detector to identify SNPs (single-nucleotide polymorphisms) and indels (insertions and deletions). The list of variants produced in a VCF format were filtered and prioritized with respect to their potential relevance for features that have been used, and a VCF file containing annotations of variant effects was produced. Finally, we filtered out variants that are unlikely to have a pathogenic effect like variants in UTRs, those upstream/downstream of the gene, deep intronic variants (but we leave variants that affect splice sites), and other non-coding variants. In addition, we filtered out variants that are common in population databases such as Gnomad. Furthermore, we prioritized variants that have been reported as pathogenic in databases like Clinvar. The aligned data were deposited in the SRA database under the accession number SRR26632668.

### 2.5 Selection of candidate variants

We focused on non-synonymous exonic variants with high correlation scores present in the affected family members and not present in the unaffected family members.

### 2.6 Primers design

Primer 3 web (https://primer3.ut.ee/) was used to design the primers listed in Table 1 below for PCR amplification and direct sanger sequencing of selected regions in the indicated genes with the identified potential mutant variants.

**TABLE 1:** The sequences and characteristics of the variants’ primers.

Gene name	Coordinates	Sequence of primer	Primer length	GC% content	Tm °C
*NEUROD1*	chr2:181,678,194-181,678,370	Forward: ACT​GGT​AGG​AGT​AGG​GGT​GT	20	55	56.9
		Reverse: ACC​TGG​TCT​CCT​TCG​TTC​AG	20	55	56.4
*IGF1R*	chr15:98,913,141-98,913,328	Forward: GAG​CCC​GGC​ATC​TTA​CTA​CA	20	55	56
		Reverse: TTG​CGA​AGA​AGT​GTG​GAT​GC	20	55	56

### 2.7 Sanger sequencing

Samples were sequenced using the BigDye™ Terminator Cycle Sequencing Kit and Applied Biosystems Genetic Analyzer according to the manufacturer’s instructions (Thermo Fisher Scientific, 2015).

### 2.8 In-silico analysis

The Mutation Mapper tool of cBioPortal was used to map mutations on proteins and their domains. The conservation of the variant locus was detected using the COBALT Alignment tool. Several prediction tools were used to predict the impact of amino acid substitution on protein structure and function, including PolyPhen-2, PROVEAN, FATHMM, SIFT, Mutation Taster, GVGD, and LIST-S2. The HaploR tool from R package was used to analyze motif changing.

### 2.9 Ethics

Ethical approval for this study was provided. A written informed consent form was obtained from all participating subjects in the study. Written informed consent was obtained from the individuals for the publication of any potentially identifiable images or data included in this article.

## 3 Results

### 3.1 Family I

Variants of the *INS, HNF1A, KCNJ11,* and *IGF1R* genes were found to have high correlation b based on data analysis. The first three variants were tested by Sanger sequencing but showed no segregation in the family because both diabetic patients and non-diabetic individuals have similar genotypes.

**FIGURE 2:**
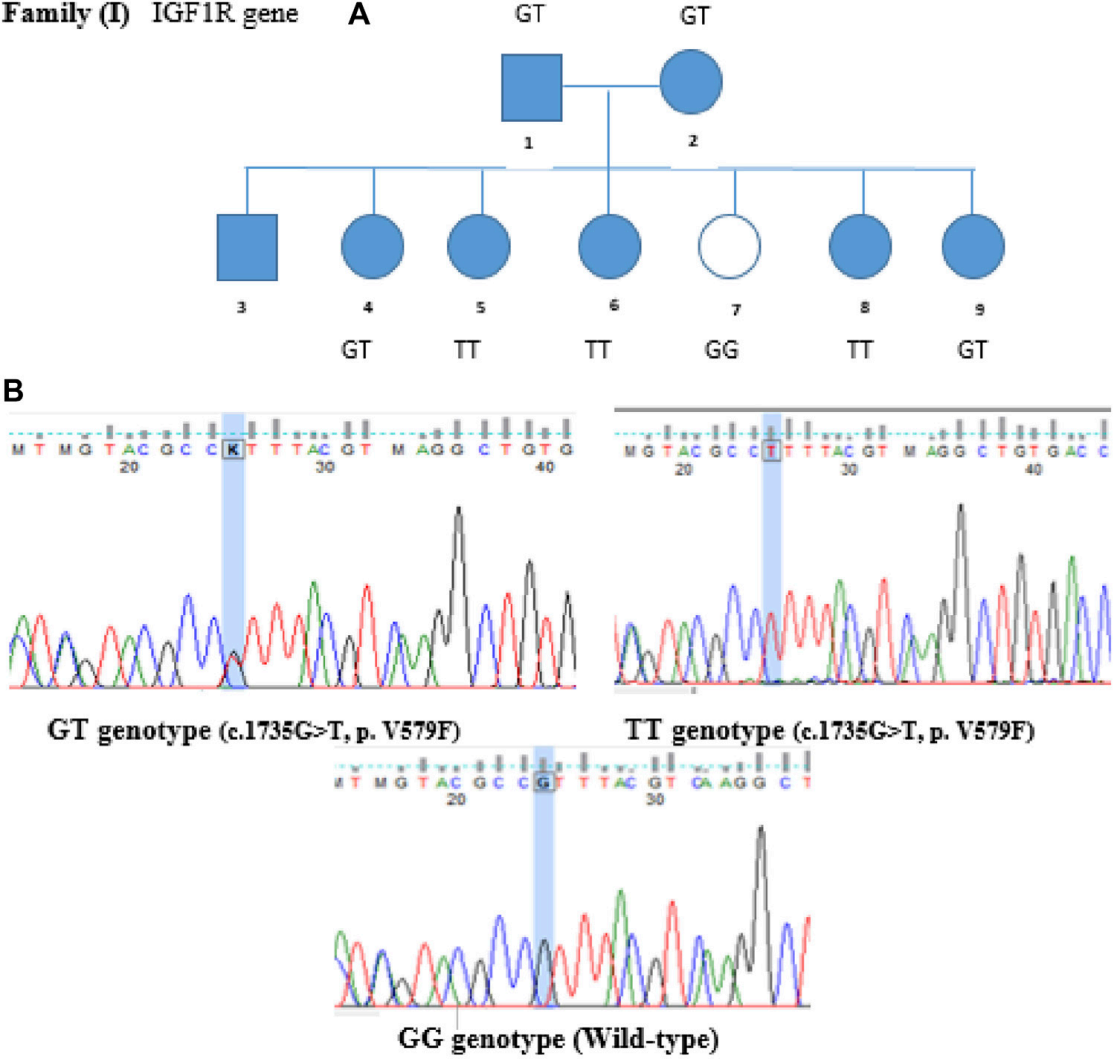
Family segregation of IGF1R variant. **(A)** Genotype results of each family member shown in the family pedigree. **(B)** Sanger Sequencing results of the detected variant in Family I.

Variants of the *IGF1R* gene were identified in T1DM patients but not in non-diabetic ones. The *IGF1R* gene is located on chromosome 15, and the identified variant was a missense mutation where Guanine is replaced by Thymidine at position 1735 (c.1735G>T), leading to replacing Valine with Phenylalanine at codon number 579. This variant was confirmed by Sanger sequencing and segregated in the family (genotypes match the phenotype). Members I-1, I-2, I-4, and I-9 are heterozygous GT, non-diabetic member I-7 is wild-type GG, and members I-5, I-6, I-8 are homozygous TT, as shown in Figure 2.

**FIGURE 3:**
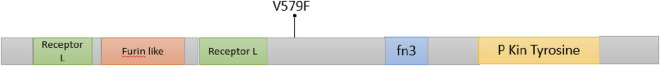
Location of IGF1R p.V579F variant on IGF1R protein.

**FIGURE 4:**
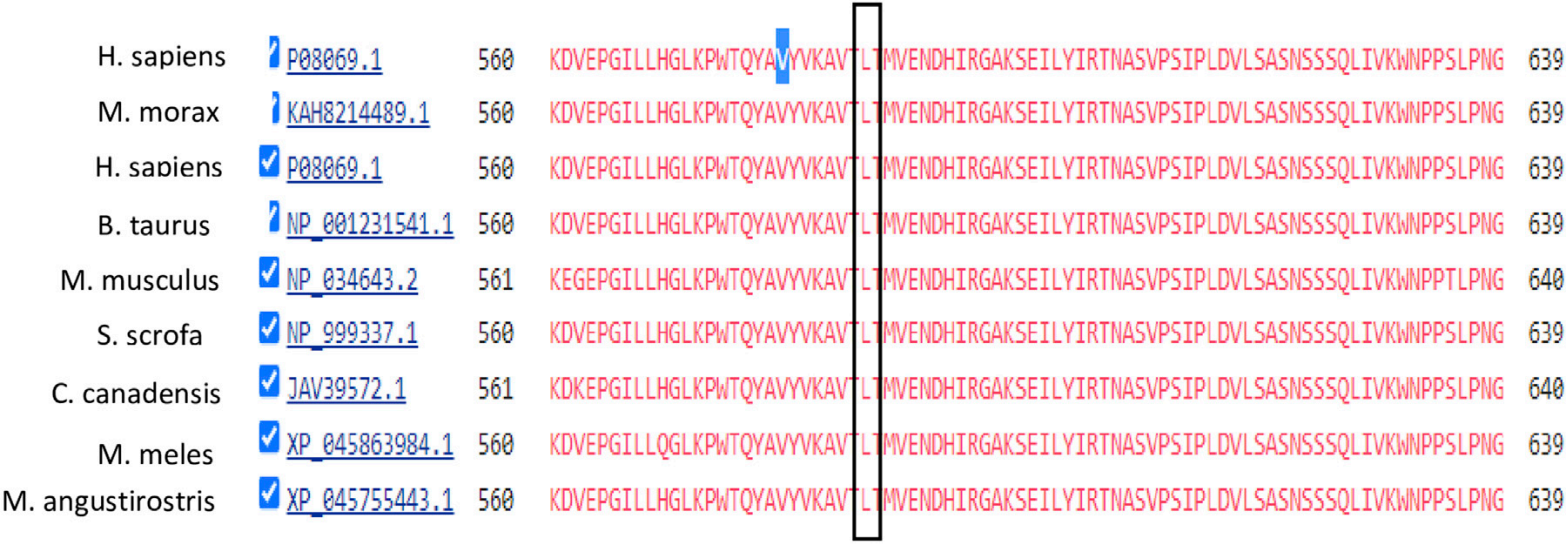
Conservation analysis of IGF1R V579 locus using COBALT alignment tool.

Mutation conservation, functional location, and pathogenicity were further investigated. The IGF1R protein is composed of five domains: two Receptor-L domains, a Furin-like cysteine-rich region, a Fibronectin type III domain, and a Protein tyrosine kinase domain. IGF1R p.V579F is located between the Receptor-L domain and Fibronectin type III domain, as shown in Figure 3. This region is highly conserved, as shown in Figure 4. Several prediction tools were used to investigate whether the IGF1R p.V579F variant has an impact on the biological function and structure of the protein. The variant is predicted to be neutral and tolerated according to PROVEAN and SIFT tools. It is also predicted to be benign by the PolyPhen-2 tool. Based on the FATHMM prediction tool, *IGF1R* p.V579F will be tolerated. However, according to the FATHMM-MKL and LIST-S2 tools, it is predicted to be damaging. The *IGF1R* p.V579F variant is disease-causing based on the Mutation Taster tool and is classified in the C45 class of Align-GVGD classes, which means it is pathogenic.

### 3.2 Family II

**FIGURE 5:**
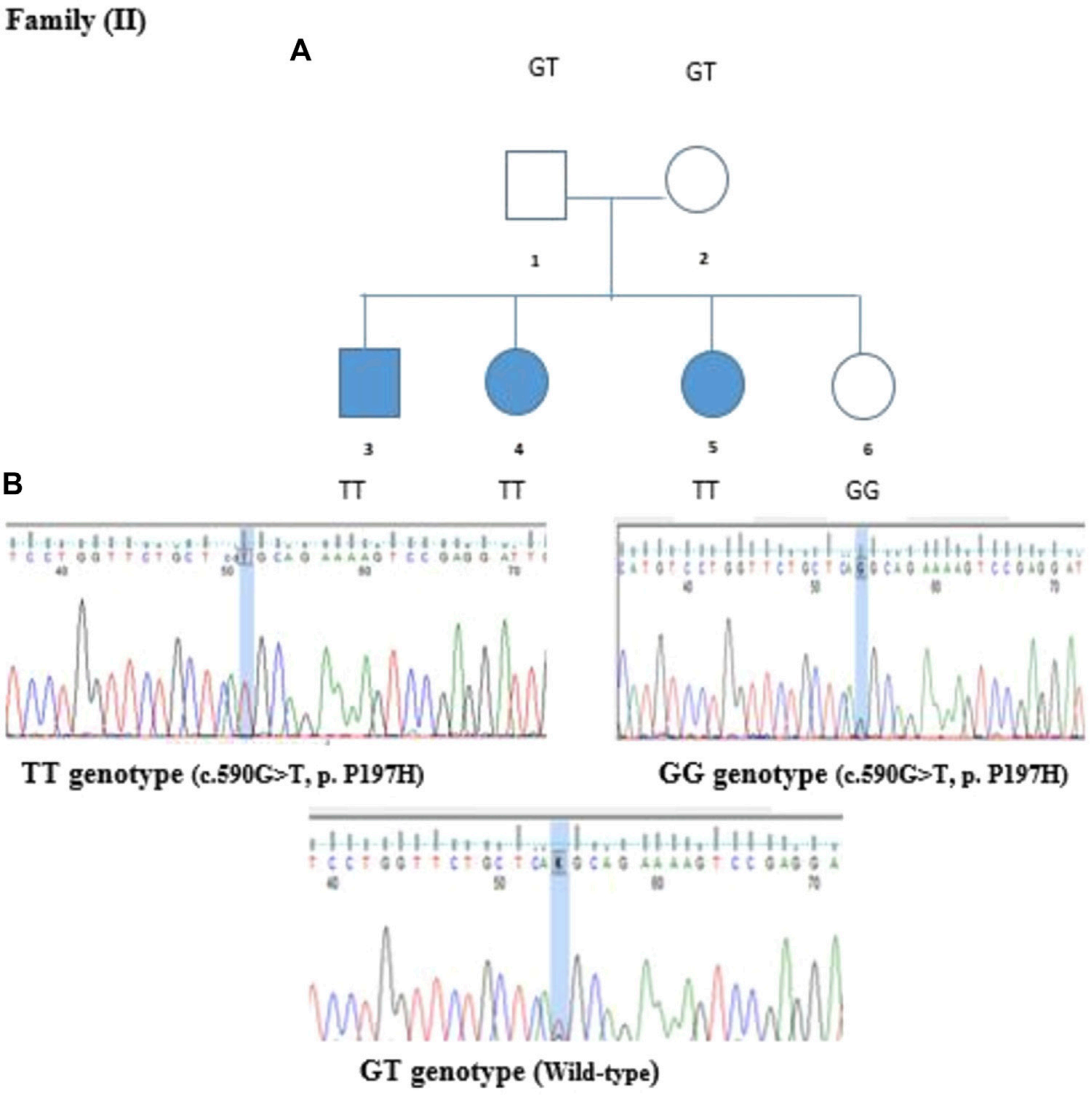
Family segregation of NEUROD1 variant. **(A)** Genotype results of each family member shown in the family pedigree. **(B)** Sanger Sequencing results of the detected variant in Family II.

In the second family, we identified variants in the *ABCC8, RYR1, CTRC*, and *NEUROD1* genes. The first three variants were tested by Sanger sequencing but showed no segregation in the family. However, a variant in the *NEUROD1* gene that is located on chromosome 2 was segregated. The identified variant c.590G>T is a missense mutation where Guanine is replaced by Thymidine at position 590 replacing Proline with Histidine. This variant was validated by Sanger sequencing and segregated in Family II, as shown in Figure 5. Members II-1 and II-2 (Parents) have heterozygous GT genotypes, whereas members II-3, II-4, and II-5 (diabetic offspring) have homozygous TT genotypes. The non-diabetic daughter (member II-6) has a wild-type GG genotype. Therefore, the segregation matches the observed expression of the disease.

**FIGURE 6:**
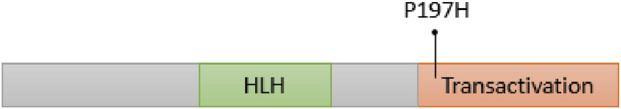
Location of NEUROD1 p.P197H variant on NEUROD1 protein.

**FIGURE 7:**
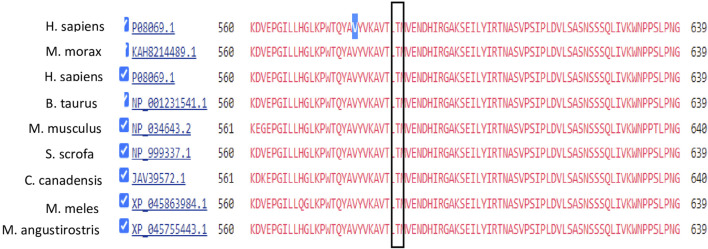
Conservation analysis of IGF1R V579 locus using COBALT alignment tool.

The *NEUROD1* protein is composed of two domains: the helix loop helix domain and the transactivation domain. The *NEUROD1* p.P197H variant is located at the transactivation domain of the *NEUROD1* protein (Figure 6) in a highly conserved region, as shown in Figure 7. The *NEUROD1* p.P197H variant is predicted to be damaging according to the PROVEAN, LIST-S2, and FATHMM-XF tools. It is also predicted to be possibly damaging according to the PolyPhen-2 tool. The variant was predicted to be disease-causing by the GVGD tool and pathogenic by the Mutation Taster tool. On the other hand, the *NEUROD1* p.P197H variant is predicted to be tolerated according to the FATHMM and FATHMM-MKL tools.

**FIGURE 8:**
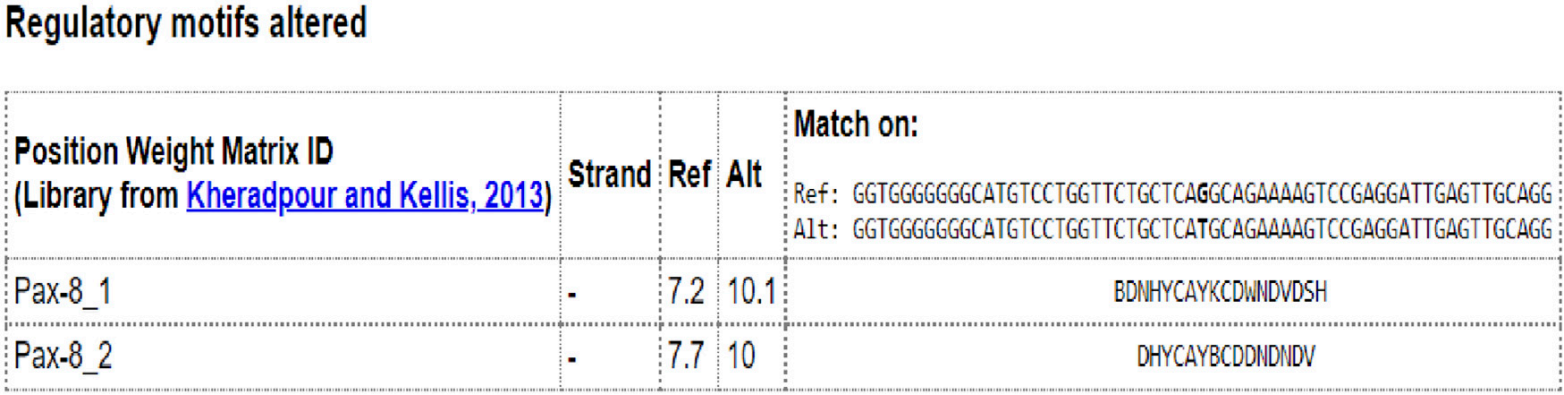
In silico analysis of NEUROD1 p.P197H variant by HaploR tool (motifs alteration).

To detect whether the *NEUROD1* p.P197H variant affects the binding motif of any transcription factor, the HaploR tool was used. Figure 8 shows the variant is located within the PAX motif.

The indicated variants in the IGF1R and NEUROD1 genes were tested in genomic DNA samples from 80 non-familial T1D patients available in our laboratory. The results showed these variants are unique mutations found in the study’s two families.

## 4 Discussion

Despite several gene mutations having been linked with type 1 diabetes mellitus, the molecular mechanisms of the disease are not fully understood. This hampers the presence of specific genetic factors that require increased efforts to unravel the transmittance of the disease within generations. This work was initiated to study two families with T1DM inherited between consecutive generations. Two putative candidate variants were prioritized and were checked for confirmation by Sanger sequencing and family segregation.

In the first family, T1D follows autosomal dominant inheritance; the IGF1R p.V579F variant was associated with T1DM in the family confirmed by Sanger sequencing and segregated in the family with the phenotype. This variant showed uncertain significance according to ACMG classification (The American College of Medical Genetics). No previous studies reported the association between this variant and T1DM or other diseases. However, the Val579 residue is highly conserved, indicating its importance in the protein with potential pathogenicity. This was confirmed with several *in silico* prediction tools, including FATHMM-MKL, LIST-S2, GVGD, and Mutation Taster, which predicted the variant to be damaging and disease-causing. Other tools predicted the variant to be benign, including PolyPhen-2, SIFT, and PROVEAN. This shows the necessity for further analysis and studies, including studies on the effects of the *IGF1R* p.V579F variant on the 3D structure of the protein and its functional implications. The *IGF1R* gene is located in chromosome 15, which encodes the insulin growth factor 1 receptor that binds with high affinity to insulin growth factor (IGF1) and with lower affinity to insulin. The structure of IGF1R and insulin receptors are very similar. Both receptors consist of a tetramer of two α subunits representing the extracellular ligand-binding domain and two β subunits representing the transmembrane domain with tyrosine kinase activity. The two subunits are linked with disulfide bonds (Hakuno and Takahashi, 2018). The IGF1 binds to the α subunits of IGF1R, causing conformational changes and stimulation of β subunit activity. This leads to autophosphorylation and transphosphorylation of tyrosine (especially tyrosine 1131, 1135, 1136, and 1221), increasing its kinase activity and thus activating and recruiting the insulin receptor substrate (IRS), CT10 regulator of kinase (CRK), and Src homology and Collagen (SHC) adaptor proteins. The latter will transduce and activate downstream signaling pathways, including the MAPK/RasRaf-Erk pathway, phosphatidylinositol-3-kinase/AKT/mTOR (PI3K/AKT) pathway, Janus kinase/signal transducer, and activator of transcription (JAK/STAT) pathway, resulting in stimulation cell proliferation, survival, and differentiation (Arnaldez and Helman, 2012). Moreover, activated AKT can activate mTOR and inhibit *FOXO1* and GSK3 downstream molecules involved in cellular processes and glucose homeostasis, resulting in gluconeogenesis inhibition and increasing glycogen and protein synthesis (Jung and Suh, 2015).

The *IGF1R* gene is highly expressed in 9% of cancer cells, including lung adenocarcinoma, cutaneous melanoma, breast cancer, and colon adenocarcinoma (Farabaugh et al., 2015). It plays an important role in phenotypic and oncogenic transformation by activating signaling pathways downstream. *IGF1R* can then interact with oncogenes such as *RAS*, *c-MYC*, and epidermal growth factor receptor (*EGFR*), causing an increase in cell migration, invasion, poor prognosis, and shorter survival rate (Sun et al., 2017).

Several studies suggest a close link between *IGF1R* function and glucose metabolism. It plays several physiological roles in glucose metabolism and tissue development including growth, heart ventricular development, neutrophils differentiation, and brain development (Wit and Walenkamp, 2013; Moody et al., 2014; Yuan et al., 2018; Jin et al., 2019). Mouse models with *IGF1R* knockout showed a significant decrease in their weight after birth. These animals suffer from reduced glucose tolerance in addition to several organ failures, leading to earlier death (Hakuno and Takahashi, 2018). The significant association between *IGF1R* expression and diabetes is evident (Fowlkes et al., 2012). One previous study found that the absence of *IGF1R* in beta cells in mice caused glucose intolerance and deficiency in insulin secretion (Xuan et al., 2002). Another study showed that the loss of one allele of *IGF1R* is associated with glucose intolerance and later insulin resistance, resulting in low birth weight and growth (Garg et al., 2011). Additionally, osteopathy defects, which are serious complications of T1DM and T2DM, result from interruption of insulin and *IGF1R* (Fowlkes et al., 2012). Noncoding RNAs (LncRNA and Micro RNAs) have a major role in disease development, including diabetes and cancer, through the regulation of *IGF1R* or other IGF signaling molecules (Chen et al., 2019). Interestingly, knockout of miR-375 in mice model revealed insulin resistance and decreased glucose homeostasis, leading to diabetes, an effect that was associated with upregulation of *IGF1R* (Kumar et al., 2021). The *IGF1R* p.V579F is predicted to be a loss-of-function mutation affecting insulin release from beta cells. The significant association between *IGF1R* and cancer results from *IGF1R* gain-of-function mutations. Therefore, diabetic patients from Family I are not expected to develop cancer due to this variant. In contrast, we cannot exclude the possibility that some *IGF1R* gain-of-function mutations are associated with diabetes as previous studies showed (Engberding et al., 2009; Kumar et al., 2021). One possible explanation is that upregulated *IGF1R* may suppress insulin signaling through a direct effect on the insulin receptor itself or its signaling mechanisms. Therefore, the collective indication of these studies and the present study indicate a significant correlation between the *IGF1R* signaling pathway and disruption in glucose metabolism leading to diabetes.

In the family with autosomal recessive inheritance, the *NEUROD1* p.P197H variant was found to be associated with T1DM. This was a missense mutation where Guanine is replaced by Thymidine at position 590 resulting in Histidine replacing Proline at codon 197. This exchange can lead to a major change in the protein structure since the variant is located in the Neuronal helix-loop-helix transactivation domain and its locus is well conserved, indicating this residue has functional significance. It is predicted to have deleterious functional consequences by several prediction tools, including PolyPhen-2, PROVEAN, SIFT, GVGD, LIST-S2, and FATHMM-XF.

The neurogenic differentiation 1 (*NEUROD1*) gene is located in chromosome 2 and encodes the basic helix-loop-helix (bHLH) transcription factor. These factors regulate the expression of genes through binding to a consensus sequence CANNTG, known as E-box. NEUROD1 combines with E2A-encoded proteins and HEB-encoded proteins. The HLH region of NEUROD1 is responsible for the dimerization between the bHLH protein and the basic region, mediating protein–DNA interactions. The *NEUROD1* gene is expressed in the intestine, pituitary gland, some central and peripheral nerves, and pancreatic islet cells. In the intestine, it stimulates the release of secretin hormone (Ray and Leiter, 2007). In the pituitary gland, it regulates the expression of proopiomelanocortin (POMC), which is a precursor of some important hormones (Parvin et al., 2017). In the nervous system, it motivates the formation of neurites, and its name is derived from this activity. This gene is also known as *BETA2* due to its function in activating the insulin gene in beta cells. Moreover, it has an important role in the development of the pancreas (Sharma et al., 1999). A previous study showed that mice lacking *NEUROD1* −/− exhibited serious complications of the pancreas, which causes a decline in beta cells, leading to severe diabetes (Naya et al., 1997).

*NEUROD1* forms a heterodimer with a bHLH transcription factor called E4, which binds to the E-box binding site in the promoter region of insulin (*INS*), glucokinase (*GCK*), sulfonylurea receptor 1 (SUR1), Paired box (*PAX*), and islet-specific glucose-6-phosphatase catalytic subunit-related protein (*IGRP*) genes and activates their expression (Rubio-Cabezas et al., 2010). These genes are known to play important roles in glucose homeostasis besides differentiation and development of pancreatic cells (Panneerselvam et al., 2019). This explains the connection between the *NEUROD1* gene and diabetes. *NEUROD1* is also involved in beta cell dysfunction during chronic hyperglycemia through two mechanisms. First, high glucose concentration will activate the expression of a small heterodimer partner (*SHP*) gene, which in turn inhibits p300-mediated pancreatic duodenal homeobox factor 1 (*PDX1*) and *NEUROD1*, resulting in insulin gene downregulation. Second**,** a decreased protein phosphatase 2 (PP2A) level will activate cyclic AMP-responsive element-binding protein (CREB), leading to *NEUROD1* and *INSULIN* repression (Horikawa and Enya, 2019; Bohuslavova et al., 2021). The detected *NEUROD1* P197H variant is found within a motif sequence specific to the PAX transcription factor. This can explain its role in developing diabetes. Further validation can be tested using CHIP-sequencing.

The *NEUROD1* gene has two exons; the first one is not translated while the second encodes for a protein with several motifs. Four variants have been identified in exon 2. One variant is p.Ala45Thr, the second one is p. Pro197His, and the third one is p.Arg111Leucine, which is located in the proximal basic portion of the basic HLH domain. The fourth variant is an insertion of cytosine in codon 206, defined as c.206 + C, leading to nonsense mutation and a premature stop codon. Malecki M. et al. studied these variants in autosomal dominant families inflicted with T2DM. The first two variants, p.Ala45Thr and p.Pro197His, were not associated with T2DM, whereas the latter two variants, p.Arg111Leu and c.206 + C, were associated with T2DM development by affecting the ability of *NEUROD1* to bind E-box and active CBP/p300, respectively (Malecki et al., 1999; Malecki et al., 2003).

**FIGURE 9:**
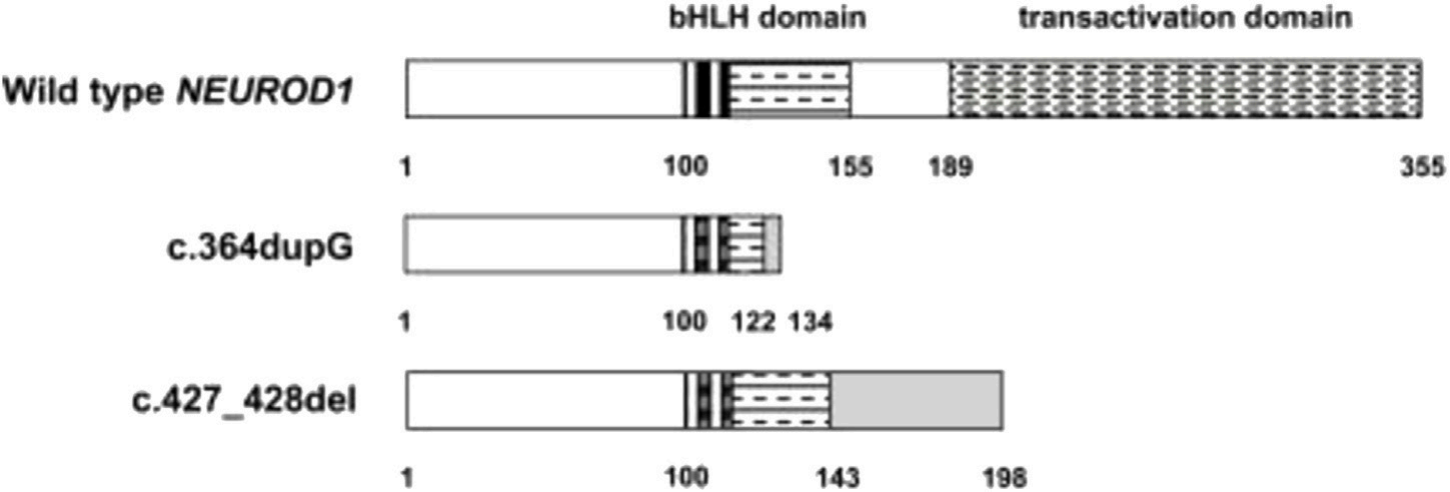
The effects of c.364dupG and c.427_428del mutations on NEUROD1 protein structure.

Previous studies reported that the *NEUROD1* p.Ala45Thr variant was associated with T1DM but not T2DM (Iwata et al., 1999; Hansen et al., 2000). In another Japanese case-control study, the researchers hypothesized that the *NEUROD*1 gene can affect the development and onset of T1DM since it acts on initial islet precursors (Yamada et al., 2001). They studied *NEUROD1* polymorphisms in 105 T1DM patients and 122 nondiabetic controls, and the diabetic patients were classified into groups according to their onset pattern. Interestingly, a significant difference in *NEUROD1* polymorphisms between cases and the control was detected in the acute-onset group (Yamada et al., 2001). This revealed the clear association of the *NEUROD1* gene with T1DM development. A study in 2010, aimed to examine the effect of *NEUROD1* mutations on patients with permanent monogenic neonatal diabetes (PNDM), found two rare homozygous mutations. The first was a duplication of a single base pair (c.364dupG), while the second was a deletion of two base pairs CT (c.427_428del)^X^. Both mutations lead to the absence of the transactivation domain from the protein due to premature truncation of the C terminus (p.Asp122Glyfs*12 and p.Leu143Alafs*55, respectively) as shown in Figure 9. These two indicated patients had some neurological abnormalities, including weakening of vision and hearing, cerebellar hypoplasia, and development delay. This leads to the conclusion that *NEUROD1* plays an important role in the pancreas as well as in the nervous system (Rubio-Cabezas et al., 2010).

In maturity-onset diabetes of the youth (MODY), several studies demonstrated some *NEUROD1* candidate mutations, including *NEUROD1* p.Pro197His, p.Asp202Glu, p.Leu157Arg, and p.Arg103Pro (Ang et al., 2016; Aǧladioǧlu et al., 2016; Szopa et al., 2016; Horikawa et al., 2018; Demirci et al., 2021). Recently, another heterozygous mutation, *NEUROD1* p.Met114Leu (c.340A > C), was reported in an Italian patient with MODY6 (Brodosi et al., 2021). This variant is predicted to be pathogenic based on many prediction tools and was later confirmed in a French family (Bouillet et al., 2020). In Latin America, a novel frameshift deletion (p.Phe256Leufs*2) in *NEUROD1* was reported in the MODY6 family (Abreu et al., 2019). Horikawa Y. and Enya H. concluded that heterozygous mutations in the *NEUROD1* gene are associated with MODY6 whereas homozygous mutations cause neonatal diabetes (Horikawa and Enya, 2019; Oliveira et al., 2020). The Diabetic members of Family II (II-2, II-3, and II-4) did not have neurological disorders. Similarly, some patients in previous studies have homozygous mutations in the *NEUROD1* gene with no neurological abnormalities (Wang et al., 2015). The reasons for this are not clear; however, one possibility suggests that the missense mutations P197H do not affect the domain needed for neurological functions.

A previous study aimed to determine if genetic variation in MODY genes can affect the response to insulin-sensitizing interventions. The study included individuals with a high risk of developing diabetes (high fasting glucose, being overweight, etc.). These subjects were divided into three groups; the first group took a placebo, the second group took a metformin drug twice a day, and the third group experienced lifestyle intervention. Different variants in several MODY genes, including *HNF4A*, *GCK*, *HNF1A, PDX1, HNF1B,* and *NEUROD1*, were genotyped in all participants. There was a significant association between *HNF4A, HNF1B*, and *NEUROD1* variants and treatment response to metformin and lifestyle intervention. One minor allele (rs6719578) in the *NEUROD1* gene showed an increase in insulin secretion in the metformin group (Billings et al., 2017). Therefore, *NEUROD1* variants can also be used in treatment programming. Its genotypes can predict the treatment response, thus it affects the selection of the drug and the doses given.

In conclusion, *NEUROD1* p.P197H and *IGF1R* p.V579F constitute high susceptibility mutations for T1DM development. Further studies should be done to confirm their causative relationship in T1DM and its mechanisms. One approach is to develop human *in vitro* modeling system(s) using selected cells in culture transformed with the mutation by CRISPR-CAS9 technology and study various responses, including glucose-stimulated insulin secretion, and the expression and activity of several genes involved in glucose metabolism. The data generated from these studies can be extended to the development of mouse models for long-term studies. These investigations will help to extend our understanding of the pathogenesis of T1D for early diagnoses and more effective clinical intervention.

## Data Availability

The original contributions presented in the study are publicly available. This data can be found here: https://www.ncbi.nlm.nih.gov/sra/SRR26632668.
